# Coronary artery calcium score—emerging role for opportunistic screening on thoracic CT and novel applications

**DOI:** 10.1093/bjr/tqag094

**Published:** 2026-04-25

**Authors:** Ryan Patel, Michelle C Williams

**Affiliations:** British Heart Foundation Centre of Research Excellence, University of Edinburgh, Edinburgh EH16SUF, United Kingdom; British Heart Foundation Centre of Research Excellence, University of Edinburgh, Edinburgh EH16SUF, United Kingdom

**Keywords:** coronary artery calcium, computed tomography, machine learning, artificial intelligence, photon counting computed tomography

## Abstract

Coronary artery calcium (CAC) can be identified on thoracic CT performed for a variety of clinical indications, including routine thoracic CT, lung cancer screening CT, and attenuation correction CT. Identification of CAC on thoracic CT as part of opportunistic screening can improve the assessment of cardiovascular risk and can identify patients who may benefit from preventative medical therapies. This approach enhances early detection of coronary artery disease within existing healthcare workflows. However, at present, there are no randomized trials that assess the clinical impact of this approach. Recently, the development of artificial intelligence/machine learning (AI/ML) models has facilitated the automated identification and quantification of CAC, offering rapid and reproducible assessments. Artificial intelligence/machine learning models could reduce radiologist workload and provide end-to-end integration into routine clinical practice. Novel technologies such as photon-counting CT offer further potential for CAC detection and risk stratification through the use of high-resolution imaging and spectral imaging with virtual non-contrast images. This review discusses the evidence for opportunistic screening for CAC on thoracic CT and highlights novel applications including AI/ML assessments, CAC as a multi-system biomarker, and the potential for photon counting CT.

## Introduction

Coronary artery disease remains the leading cause of morbidity and mortality worldwide, accounting for more than 19.8 million deaths each year.^1^ Despite major advances in treatment and prevention, many individuals continue to present with myocardial infarction or sudden cardiac death as the first manifestation of coronary artery disease. This highlights the ongoing need for accurate methods to identify high-risk individuals before the development of symptoms. The current approach to risk stratification for coronary artery disease in asymptomatic patients relies on probabilistic risk scores, which may over or underestimate the true burden of disease. Opportunistic assessment of coronary artery calcium (CAC) provides a direct imaging marker of atherosclerosis, enabling identification of existing coronary artery disease rather than simply estimating its likelihood.

Coronary artery calcium is a robust imaging biomarker for the presence and severity of atherosclerosis in the coronary arteries. Calcification is one component of atherosclerotic plaque, which also contains non-calcified plaque including fibrosis, lipid, and necrotic components. Coronary artery calcium can be quantified using CT, most commonly through non-contrast electrocardiogram (ECG) gated cardiac CT. Over the past 3 decades, large population studies and clinical trials have established the prognostic value of CAC, with higher CAC associated with increased rates of cardiovascular events and mortality in symptomatic and asymptomatic patients.^2^^,^^3^ However, CAC can be identified and quantified on non-gated CT of the chest, including both non-contrast and contrast-enhanced images (Figure 1). It can also be identified and quantified on low radiation dose images such as lung cancer screening CT and CT performed for attenuation correction during nuclear imaging studies (Figure 2). This offers the possibility of opportunistic screening for coronary artery disease, where CAC is identified on CT scans performed for other clinical indications. This is potentially a cost-effective, widely accessible option for the identification of coronary artery disease.

**Figure 1:**
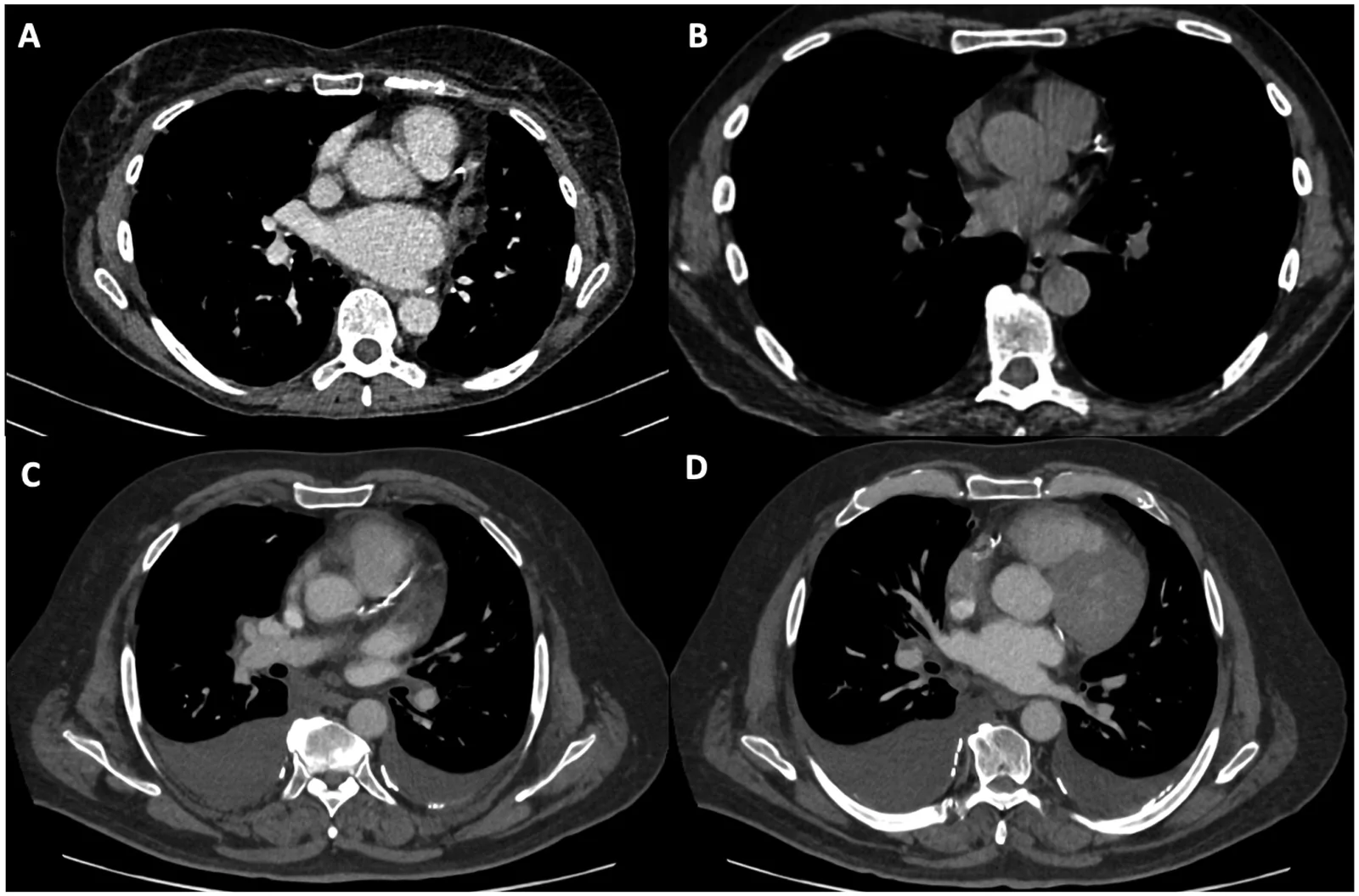
Coronary artery calcium (CAC) identified on contrast-enhanced and non-contrast CT in 3 patients. (A) Mild CAC in the mid left anterior descending artery (LAD) in a patient undergoing follow-up CT for metastatic breast cancer. (B) Moderate CAC in a patient undergoing non-contrast CT for the assessment of cough. (C, D) Severe CAC in the left anterior descending coronary artery, left circumflex, and right coronary artery in a patient with bilateral pleural effusions secondary to cardiac failure.

**Figure 2:**
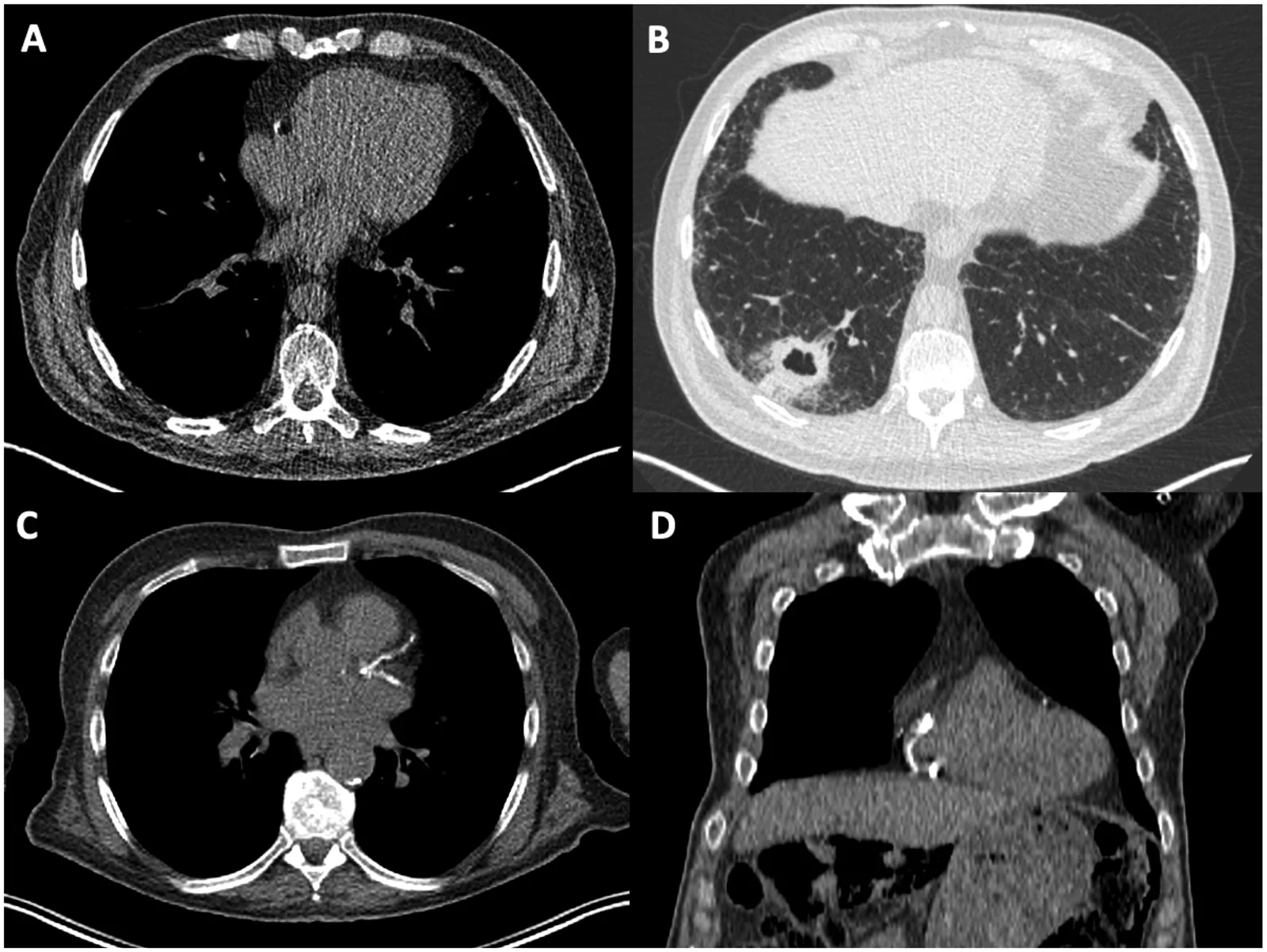
Coronary artery calcium (CAC) identified on low-dose CT performed for lung cancer screening (A, B) and low-dose CT performed for attenuation correction for PET (C, D). Mild coronary artery calcification (A) identified in the right coronary artery of a patient with (B) right lower lobe lung cancer. Severe coronary artery calcification in the left coronary artery, left circumflex artery (C), and right coronary artery (D) in a patient undergoing PET/CT for cardiovascular assessment.

Given the large and rapidly growing volume of CT performed worldwide, the automated extraction of CAC information could provide an opportunity to assess cardiovascular risk without additional investigations or radiation exposure. Recent advances in artificial intelligence/machine learning (AI/ML) further enhance the feasibility of opportunistic screening without adding to the radiologist workload. Coronary artery calcium has also emerged as a potential biomarker of multi-system disease and biological aging. In parallel, novel CT technologies such as photon-counting CT (PCCT) and spectral reconstructions are providing new ways to identify and quantify CAC. This review summarizes the rational and evidence for opportunistic screening with CAC and highlights recent novel applications.

## CT assessment of coronary artery calcification

Coronary artery calcium reflects the deposition of calcification within the coronary artery wall as part of the atherosclerotic disease process. Calcification typically develops later in the natural history of plaque development and progression, which involves cycles of vascular injury and healing. Coronary artery calcium correlates with the total burden of atherosclerosis but does not directly measure higher risk plaque components such as non-calcified and low attenuation plaque. Thus, CAC should be considered a surrogate marker of coronary artery disease burden.

The assessment of CAC with CT was first established in the early 1990s, initially with electron beam CT.^4^ With advances in multi-detector CT and ECG gating, CAC scoring became highly reproducible and more widely applicable. Dedicated, non-contrast ECG-gated CT remains the reference standard for CAC measurement, offering rapid acquisition, low radiation dose, and excellent reproducibility. Standardized acquisition protocols have been developed for dedicated cardiac CT. However, opportunistic CAC assessment may be performed on a wide range of non-gated chest CT examinations acquired for other clinical indications. This introduces potential variability related to differences in scanner models, vendors, reconstruction algorithms, slice thickness, and acquisition parameters. These factors can influence the identification and quantification of CAC and should therefore be considered when interpreting opportunistic assessments, particularly when comparing heterogenous CT platforms or across serial examinations.

Several methods have been developed for the quantification of CAC on CT (Table 1). The most widely used method for ECG-gated CT is the Agatston score that calculates a weighted sum of the area of calcified lesions, adjusted for their peak density.^4^ Other quantitative methods include the calcium volume score and the calcium mass score, which are not routinely used in clinical practice. Alternatively, a visual ordinal score can be applied without the need for additional software, by classifying the severity of coronary artery calcification as none, mild, moderate, or severe, and summarized on a per segment, per vessel, or per patient basis. For assessment of CAC on thoracic CT, the British Society of Cardiovascular Imaging, British Society of Cardiovascular Computed Tomography, and British Society of Thoracic Imaging guidelines^5^ recommend the use of a visual per patient score which can easily be applied across all CT imaging types and does not require additional software. This guideline recommends the reporting CAC on a per patient level as mild, moderate, or severe, with the presence of any CAC being an indication for reviewing modifiable cardiovascular risk factors and managing them as per existing primary prevention guidelines. The identification of CAC in an asymptomatic patient is not an indication for additional imaging tests. The Coronary Artery Calcium Data and Reporting System (CAC-DRS) was developed by the Society of Cardiovascular Computed Tomography to standardize the reporting of CAC on non-contrast CT performed with or without ECG gating and is used in some centers to provide standardized reporting and management recommendations.^6^

**Table 1:** Methods to quantify coronary artery calcification on CT.

Method	Description	Strengths	Limitations	Recommended use
**Agatston score**	Weighted sum of calcified lesions ≥130 HU, adjusted for the peak density within the lesion	Widely used with a large evidence base	Sensitive to acquisition and reconstruction parameters. Less reproducible at low scores. Requires dedicated software	Routinely used for ECG-gated CT
**Calcium volume score**	Quantifies the volume of lesions ≥130 HU	Less affected by slice thickness and reconstruction	Does not include assessment of density. Less evidence base. Requires dedicated software	Not routinely used
**Calcium mass score**	Accounts for both lesion volume and mean density	Incorporates size and density	Requires scanner specific calibration factor. Less evidence base. Requires dedicated software	Not routinely used
**Visual ordinal score—per vessel or segment**	Semi-quantitative visual grading of vessels or segments which is summed	Simple and easy to apply. Does not require dedicated software	Less accurate. Potential observer variability	Not routinely used
**Visual ordinal score—per-patient**	Semi-quantitative visual grading (none, mild, moderate, severe)	Simple and easy to apply. Does not require dedicated software. Feasible on non-gated CT and contrast and non-contrast CT	Less accurate. Potential observer variability	Routinely used for non-gated thoracic CT including lung cancer screening CT
**CAC-DRS (coronary artery calcium data and reporting system)**	Per-patient summary of total calcium score (Agatston score or visual assessment) and number of involved arteries	Summarizes information and provides associated management recommendations	Requires referrers to understand the scoring system	Used in some centers

There is an extensive body of literature showing that CAC burden on ECG-gated CT is a powerful predictor of major adverse cardiovascular events (MACEs), including myocardial infarction and cardiovascular death.^2^^,^^7–9^ A CAC score of zero from ECG-gated CT is associated with a very low short- and medium-term event rate^10^^,^^11^ and higher CAC score categories are associated with stepwise increase in risk. Notably, CAC scoring on ECG-gated CT improves classification of patients from low to intermediate risk,^12^^,^^13^ identifying those who may benefit from more intensive preventative therapies. Given its robust prognostic value, CAC scoring with ECG-gated CT has been incorporated into international guidelines for coronary artery disease prevention and management.^14^^,^^15^ For asymptomatic patients CAC can reclassify cardiovascular risk from traditional probabilistic scores and may be useful in patients with borderline risk scores.^14^ For patients with symptoms of suspected coronary artery disease, the presence of coronary artery calcification can be used to adjust the estimated clinical likelihood of obstructive coronary artery disease and guide further investigations.^15^

## Opportunistic screening for coronary artery disease

Opportunistic screening refers to the detection of disease or risk factors using examinations that are performed for a different primary purpose. CT-based opportunistic screening can identify unexpected pathologies or risk factors and provide additional value for patients and healthcare providers without additional radiation exposure or patient time.^16^ Current approaches for identifying individuals at risk of coronary artery disease rely on the use of cardiovascular risk scores or the presence of symptoms. However, many patients present with advanced coronary artery disease, acute coronary syndrome, or sudden cardiac death without having prior symptoms.^17^ In addition, cardiovascular risk scores may underestimate disease in some patients, particularly younger women, and overestimate disease in others, particularly older men.^18^ There is therefore a need for strategies that can identify high-risk individuals earlier and more accurately. There is a large potential for impact as, for example, in the United States in 2023 there were ∼24.5 million CT performed which covered the chest (including whole body CT, excluding cardiac CT) and ∼30 million CT abdomen and pelvis which also normally covers the lower aspect of the heart.^19^

Coronary artery calcium can be identified on any CT scan that covers some or all the heart. This includes contrast-enhanced and non-contrast CT of the chest and abdomen. Coronary artery calcium can also be identified on low-dose CT performed for lung cancer screening and attenuation correction CT performed as part of PET and SPECT (Figure 2). Despite technical limitations that are discussed later in this manuscript, CAC can be identified and quantified on non-dedicated CT with good diagnostic accuracy (Figure 3) compared to ECG-gated CT^20^ and with good inter- and intra-observer agreement.^21^ However, motion artifact and contrast opacification can mean that CAC can be missed or underestimated on routine thoracic CT. A meta-analysis of 15 studies showed that CAC assessed on non-gated CT had an excelled agreement with ECG-gated CT, with a pooled correlation coefficient of 0.96 (95% CI, 0.92 to 0.98, *P* < .001).^22^ Therefore, thoracic CT offers a significant potential for opportunistic screening for coronary artery disease, enabling early detection and intervention.

**Figure 3:**
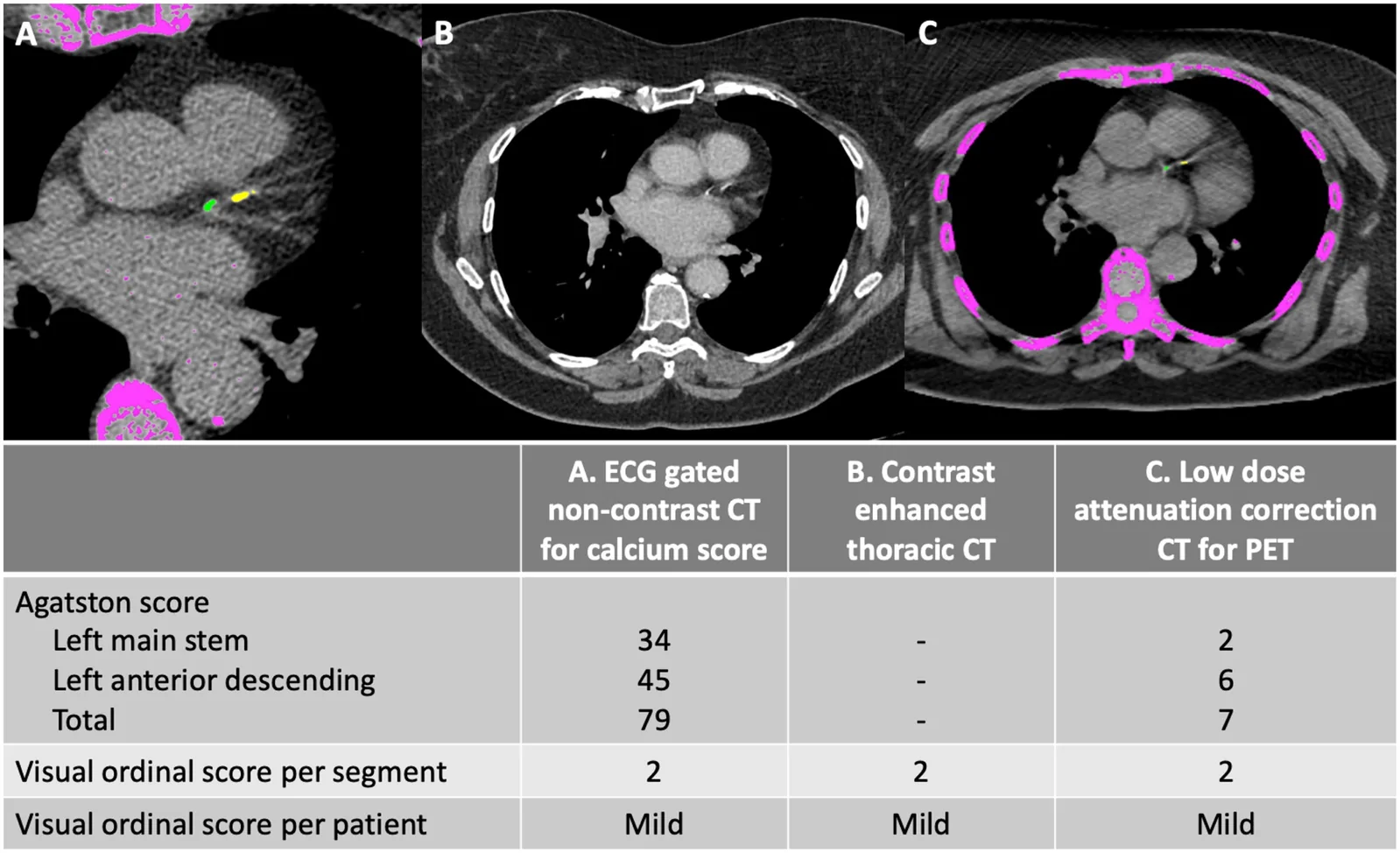
Coronary artery calcification on (A) electrocardiogram gated CT, (B) contrast-enhanced thoracic CT performed 1 months later, and (C) low-dose attenuation correction CT performed during PET 1 week later. Coronary artery calcium was assessed quantitatively using the Agatston score and with visual ordinal scores on a per segment and per patient basis. Abbreviation: ECG = electrocardiogram.

## Clinical benefits of opportunistic CAC screening for coronary artery disease

Many patients undergoing thoracic CT for non-cardiac indications, such as the assessment of pulmonary disease, oncological staging scans, or lung cancer screening, have significant CAC.^22^ Reporting CAC in these asymptomatic patients may identify the presence of previously unknown coronary artery disease. Coronary artery calcium is a common finding on thoracic CT, with a pooled prevalence of 52% (95% CI, 46% to 58%) in a meta-analysis of 94 studies including 89 006 patients.^22^ There was moderate heterogeneity between the studies included in this meta-analysis and higher CAC prevalence was found in studies of older patients and those with a higher prevalence of males. Additional sources of heterogeneity could include the indication for imaging and CT acquisition parameters, such as the inclusion of lung cancer CT.

From a prognostic perspective, incidental CAC on thoracic CT is a robust and independent predictor of adverse cardiovascular outcomes, including all-cause mortality and MACEs.^22^ A meta-analysis of 51 582 patients who were followed up for 51.6 ± 27.4 months showed that patients with CAC had an increased risk of all-cause mortality with a pooled relative risk of 2.13 (95% CI, 1.57 to 2.90, *P* = .004) and of MACE with a pooled relative risk 2.61 (95% CI, 2.17 to 3.74, *P* < .001).^22^ Coronary artery calcium on thoracic CT is associated with increased risk of cardiovascular morbidity and mortality including myocardial infarction and composite endpoints such as revascularization, stroke, limb ischemia, sudden cardiac death, and heart failure.^23^ In patients with COVID-19, the presence of CAC was associated with increased in-hospital mortality.^24^ Interestingly, incidental CAC has been shown to be a more important predictor of mortality than the severity of pulmonary embolism in patients undergoing CT pulmonary angiography,^25^ highlighting the importance of CAC assessment alongside treatment of the primary diagnosis.

Around the world large numbers of patients are currently undergoing low-dose thoracic CT as part of lung cancer screening programs. There are important overlaps between risk factors for coronary artery disease and lung cancer, such as smoking. Therefore, integration of CAC assessment into lung cancer screening could provide the opportunity to identify both cancer and cardiovascular disease, the 2 leading causes of death worldwide. The prevalence of CAC on lung cancer screening CT ranges from 14.8% to 98% among different research cohorts.^26^ Among patients undergoing lung cancer screening a meta-analysis of 5 studies identified that patients with CAC were at increased risk of MACE and all-cause mortality, with a pooled relative risk of 3.27 (95% CI, 1.88 to 5.68, *P* = .004).^22^ Incidental CAC on CT performed for lung cancer screening has been shown to have long-term prognostic implications, extending to 25 years.^27^ In addition, modeling performed in a Dutch health technology assessment found that combined screening for lung cancer, chronic obstructive pulmonary disease, and cardiovascular disease would be more cost effective than screening for lung cancer alone.^28^

Identification of incidental CAC could also lead to changes in management, such as the commencement of statin therapy, improvement in blood pressure control, and lifestyle modification such as smoking cessation, dietary improvements, and increased physical activity. On non-gated thoracic CT, the presence of CAC can reclassify patients whose risk may have been overestimated or underestimated by traditional risk factor assessments.^21^

A meta-analysis of 7 studies found that reporting incidental CAC on thoracic CT was associated with increased prescriptions for statin therapy in 5 studies (71%).^23^ The Incidental Coronary Calcification Quality Improvement Project (NOTIFY-1) showed that patients who were randomized to being informed of incidental CAC on thoracic CT were more likely to be receiving statin therapy at 6 months (51.2% versus 6.9%, *P* < .001) and had lower low-density lipoprotein cholesterol levels compared to patients who were randomized to standard care.^29^ In addition, they found that there was an increase in primary care physician encounters, but not cardiology clinic appointments, and an increase in the use of non-invasive stress testing (11.6% versus 2.3%, *P* = .018) but not invasive coronary angiography.^29^

There is extensive observational data showing the prognostic impact of CAC in cohort studies, and randomized studies that show that CAC can influence treatment endpoints and behavior change. However, randomized studies of hard cardiovascular outcomes have not yet been published. We are awaiting the results of randomized studies that show that changing management based on this information improves prognosis. The NOTIFY 2: Picture of Incidental Calcium to Understand Risk Estimate (PICTURE, NCT05588895) study will assess the clinical impact of randomization to notification of incidental CAC on thoracic CT or standard care on 10-year cardiovascular events. In addition, a large, randomized trial has evaluated the impact of screening for CAC using dedicated ECG-gated CT on management changes. The Risk Or Benefit IN Screening for CArdiovascular disease (ROBINSCA) trial randomized 43 447 patients to either screening for cardiovascular disease with traditional risk factor assessment or with ECG-gated CT for assessment of CAC.^30^ The ROBINSCA trial showed that screening with CAC reduced the total number of individuals who were recommended to take preventative medication^31^ and that these patients were more likely to be compliant with taking blood pressure and cholesterol lowering medications after 14 months of follow-up.^30^ There was no impact on quality of life or clinically relevant anxiety levels with CAC-based screening compared to traditional risk score-based screening in the ROBINSCA trial.^32^ Participants informed of a high CAC score reported slightly higher anxiety levels than those with a low CAC score or risk score results, but this difference did not reach the threshold for a clinically meaningful change.^32^ The long-term prognostic impact of these management changes remain to be determined in the ROBINSCA trial with the cardiovascular endpoint results awaited.

## Challenges for opportunistic screening with CAC for coronary artery disease

Despite the growing enthusiasm for opportunistic identification of CAC on thoracic CT, there are several challenges that should be considered. This includes the impact of image acquisition and reconstruction factors on the identification and classification of CAC, variability in reporting methods, integration into clinical workflows, and the need for randomized trials and cost-effectiveness analyses to support its delivery.

Without ECG gating and with different acquisition parameters (scanner vendor, tube voltage, slice thickness, reconstruction method, etc.), thoracic CT can under or overestimate the presence and severity of CAC compared to ECG-gated CT. Standardized ECG-gated CT is performed with 3 mm non-overlapping slices, with a tube voltage of 120 kV and a filtered back projection reconstruction algorithm. For thoracic CT, the use of thinner slices, lower tube voltages, iterative and other reconstruction algorithms, and no ECG gating can all impact the detection and quantification of calcification. Therefore, the absence of CAC on thoracic CT may not mean the absence of coronary artery disease. Iodinated contrast may mask the presence of CAC, which is more easily seen on non-contrast CT. The impact of this may be small, particularly if CAC is considered in broad categorical groups, but for patients with only a small amount of CAC, this could make the difference between recommendations for lifelong medical therapy or no therapy.

Another important challenge is how the assessment of CAC is incorporated into clinical workflows. From a health system perspective, an important question remains around cost-effectiveness. While modeling studies suggest that combined lung cancer and cardiovascular screening could be cost-effective,^33^ evidence from randomized controlled trials is not currently available. Concerns have been raised regarding implications on radiologist workload and potential downstream “unnecessary” investigations. The NOTIFY-1 trial found that within the notification arm, there was a small increase in the number of patients undergoing additional CAD testing, mainly in the form of cardiac stress tests.^29^ Integration of opportunistic CAC screening into clinical practice therefore requires careful consideration of resource allocation.

Clear guidance for referring clinicians on how to interpret and act on CAC results is important, as is described in the BSCI/BSCCT/BSTI guidelines.^5^ Guidelines for the reporting of CAC on thoracic CT have also been produced by other societies including the Society of Cardiovascular Computed Tomography/Society of Thoracic Radiology,^34^ American College of Radiology,^35^ and National Lipid Association.^36^ For lung cancer screening, the European Respiratory Society, European Society of Thoracic Surgeons, European Society for Radiation Oncology, European Society of Radiology, European Society of Thoracic Imaging, and European Federation of Organisations for Medical Physics joint statement on management of incidental findings recommends reporting CAC on low-dose CT screening for lung cancer.^37^ There may be uncertainty among referring clinicians on who will act on these results, particularly where there is crossover between primary and secondary care. Clear local guidelines and structured pathways may be beneficial to ensure that management is consistent and opportunities for coronary artery disease prevention are not missed. Most patients will be asymptomatic and only require assessment of their cardiovascular risk factors. However, a small number of patients who have previously unrecognized symptoms of suspected coronary artery disease will require referral to the appropriate chest pain or cardiology services and may require further investigations. How this information should be used within the context of the patient’s overall clinical situation and cardiovascular risk should be considered by the referring clinician. There may be patients where information on CAC does not provide additive value, for example, those with a life expectancy of less than 6 months. However, a diagnosis of cancer does not mean that information on CAC can always be ignored, as today many patients with CAC survive their initial cancer treatments and at a later time experience cardiovascular events.^38^

It is important to develop methods where there is equitable access to information on CAC for all patients. At present, there is a high variability in the reporting of CAC on thoracic CT, varying between 18% and 93% in a recent meta-analysis.^22^ A survey of British radiologists found that only 11% routinely reported calcification on thoracic CT^39^ and a survey of American College of Radiology members found that only 32% of cardiac radiologists and 17% of non-cardiac radiologists were aware of the correlation between qualitative and quantitative CAC scores.^40^ Implementation of an education program for reporting radiologists was effective in improving assessment of CAC on thoracic CT^41^ and such endeavors are essential for the implementation of standardized practices, ensuring consistent and accurate reporting across diverse clinical settings.

## Novel applications

The use of AI/ML models to identify CAC can provide rapid, accurate, and reproducible assessments with the potential to reduce radiologist workload (Figure 4).^42^ Multiple AI/ML models have been developed to identify and quantify CAC on ECG-gated CT and AI/ML models have been further trained to deal with thoracic CT with a range of imaging protocols.^43–46^ Some of these are commercially available and are included on the FDA “AI-enabled Medical Devices List” (https://www.fda.gov/medical-devices/software-medical-device-samd/artificial-intelligence-enabled-medical-devices). However, it must be remembered that there are also many limitations to the use of AI/ML in clinical practice including biases, generalizability, clinical validation, interoperability, data privacy, cost effectiveness, post market surveillance, and regulation.^42^

**Figure 4:**
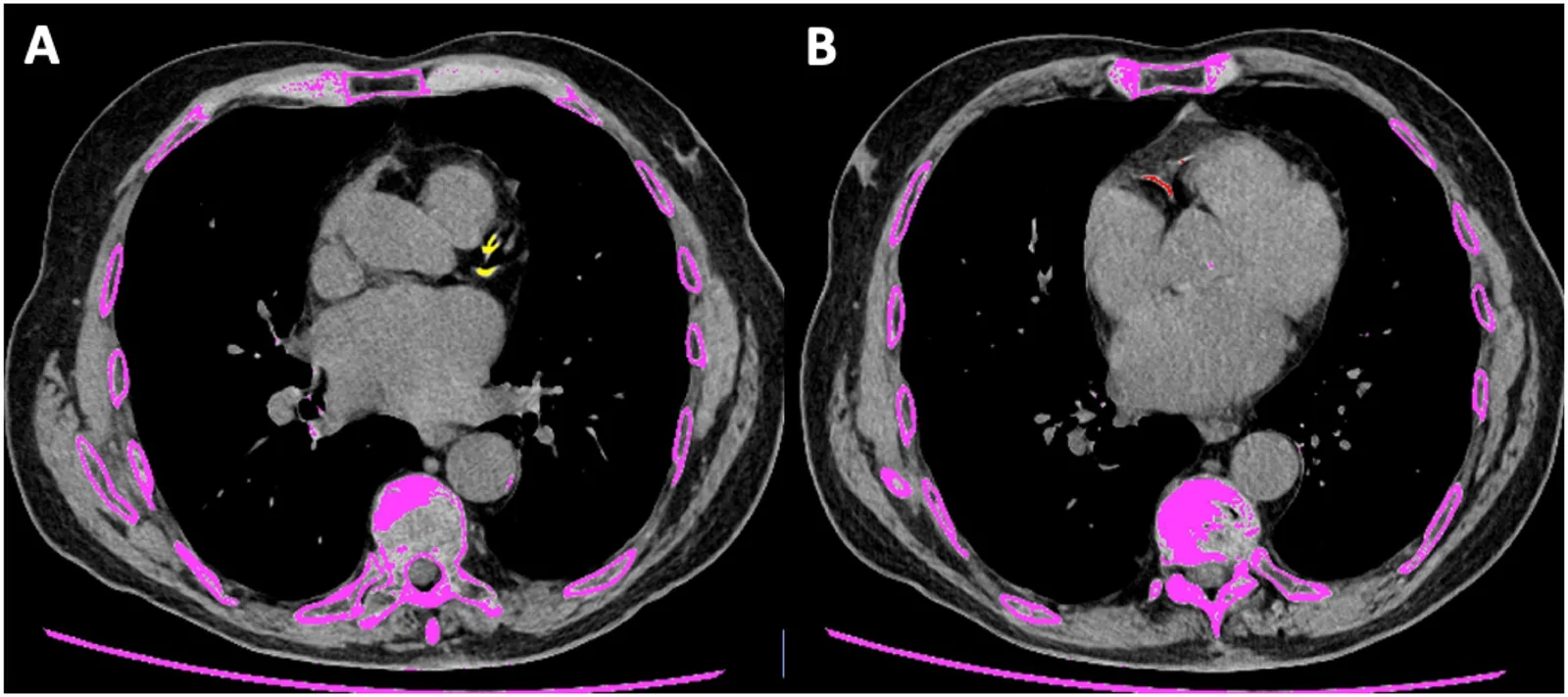
Coronary artery calcification identified on thoracic CT assessed using a machine learning model. Coronary artery calcification in the (A) left anterior descending coronary artery and ramus branch (yellow) which was correctly identified. Coronary artery calcification in the right coronary artery was correctly identified but its volume was overestimated due to motion artifact (B, red). The total Agatston coronary artery calcium score was 327 Agatston units. Pixels above 130 Hounsfield units are highlighted by the software in pink.

Several of these AI/ML models have been assessed for their prognostic implications. Using gated and non-gated CT from the Framingham Heart Study, National Lung Screening Trial (NLST), PROMISE, and ROMICAT-II, Zeleznik et al^43^ trained a deep learning model for the assessment of CAC on thoracic CT and showed that it had good agreement with manual assessments and could identify patients undergoing lung cancer screening who were at increased risk of cardiovascular death. This AI/ML model identified that patients with a high calcium score (>300 Agatston units) were nearly 4 times more likely to experience cardiovascular death compared to patients with no CAC after 6.7 years of follow-up, adjusted for age, sex, diabetes, heart disease, and hypertension (HR 3.87, 95% CI, 2.45 to 6.11, *P* < .001).^43^ Hagopian et al^45^ trained a deep learning model to assess CAC using data from the Veterans Affairs system in the United States. Their AI/ML model showed that patients with a CAC score >400 Agatston units were more likely to experience 10 year all-cause mortality (HR 3.49, *P* < .0005 compared to patients with no CAC) and the composite endpoint of stroke, myocardial infarction, and death (HR 3.00, *P* < .005 compared to patients with no CAC).^45^ Among 8052 patients undergoing low-dose non-gated CT for lung cancer screening, they identified CAC in 79% of patients and that 30.6% of patients with a CAC score >400 Agatston units were not taking lipid lowering therapies.^45^ Together these studies show that AI/ML models can effectively predict cardiovascular risk and highlight gaps in preventative care.

Once CAC has been identified, further AI/ML models could be used to optimize patient care. For example, natural language processing and large language models could be used to identify the presence of CAC from radiology reports and to assess whether a patient is already on appropriate preventative medication. This could be used to target appropriate interventions to patients who might benefit from them, making healthcare more personalized and cost-effective (Figure 5). Such multi-step or agentic AI/ML models are not yet available for clinical use.

**Figure 5:**
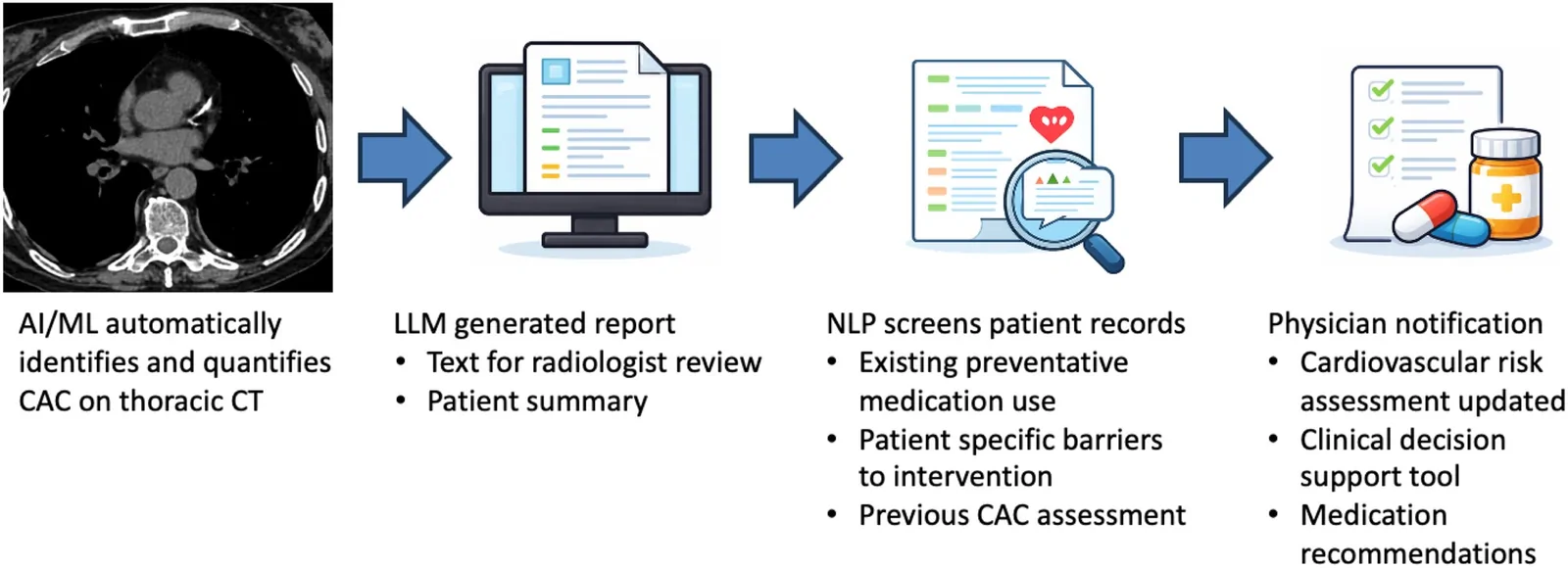
A proposed end-to-end artificial intelligence/machine learning (AI/ML) pipeline for opportunistic coronary artery calcium (CAC) assessment and appropriate clinical action. Artificial intelligence/machine learning algorithms can automatically identify and quantify CAC on thoracic CT performed for a wide variety of non-cardiac indications. The results can be incorporated into a large language model generated report providing structured text for radiologists to review and approve, along with a concise patient summary. Natural language processing (NLP) models can then screen the patient’s electronic health records to identify current preventative medication use, prior CAC assessment and patient specific barriers to intervention. Finally, clinicians can be notified of any patient that would benefit from additional assessment with the CAC information incorporated into cardiovascular risk assessment, and appropriate changes in management or medication recommendations supported by clinical decision support tools. Abbreviation: CAC = coronary artery calcification.

Several groups have attempted to go beyond the traditional methods of quantifying CAC such as the Agatston scores, to incorporate aspects of density, distribution, and radiomic characteristics. Radiomics is an evolving field that describes the process of extracting many quantitative features from medical imaging to capture underlying tissue characteristics such as texture, shape, and spatial distribution, going beyond the level that can be ascertained by the human eye. Radiomic characteristics have been shown to identify high-risk plaque characteristics on coronary CT angiography^47^ and improve the prediction of myocardial infarction by coronary CT angiography, beyond clinical factors and plaque burden.^48^ Calcium radiomic characteristics have been shown to be additive to the coronary artery calcium score to predict MACE at 9.1 years.^49^ On non-gated thoracic CT, the assessment of coronary radiomic characteristics will be challenging due to motion artifact and variability in image acquisition parameters. Nevertheless, on low-dose chest CT from the Multi-Ethnic Study of Atherosclerosis (MESA) study, the radiomic characteristics of the entire heart, including areas of coronary artery calcification, could predict the presence of severe coronary artery stenosis better than cardiovascular risk factors alone.^50^ These radiomic assessments of CAC are not yet ready for clinical practice. Challenges of implementing radiomic assessment include variability between acquisition protocols, scanners, manufacturers, and reconstruction parameters. Tools for image harmonization before or after radiomic assessment are being developed, including statistical and AI/ML approaches. However, extensive further validation and standardization is required before radiomic characteristics can be reliably integrated into routine clinical workflows, with particular attention required to consistency and accuracy across diverse imaging datasets.

Recently, the development of photon counting CT (PCCT) has improved our ability to assess small structures such as the coronary arteries. Photon counting CT uses detectors that directly count the number and energy of photons, which gives us the ability to perform higher resolution imaging and spectral material decomposition analysis. Higher resolution imaging would reduce calcium blooming artifacts that can lead to overestimation of calcification and can enable the identification of smaller areas of calcification than are possible with energy integrated detector CT. However, standardized calcium scoring acquisitions are performed with 3 mm non-overlapping slices, so consensus will be required regarding how to use this additional information on smaller areas of atherosclerosis in clinical practice. If a 3 mm slice thickness is used, then early studies have shown that PCCT leads to lower CAC scores than standard energy integrated detector CT.^51^ From the contrast-enhanced PCCT multi-energy spectral imaging, it is possible to reconstruct virtual non-contrast (VNC) images on which CAC can be assessed. Coronary artery calcium scores from VNC images have been shown to be slightly lower than those from true non-contrast images.^52^ The use of VNC images from PCCT could enable the assessment of CAC scores on contrast-enhanced CT (Figure 6) but these reconstructions are not currently recommended for clinical use.

**Figure 6:**
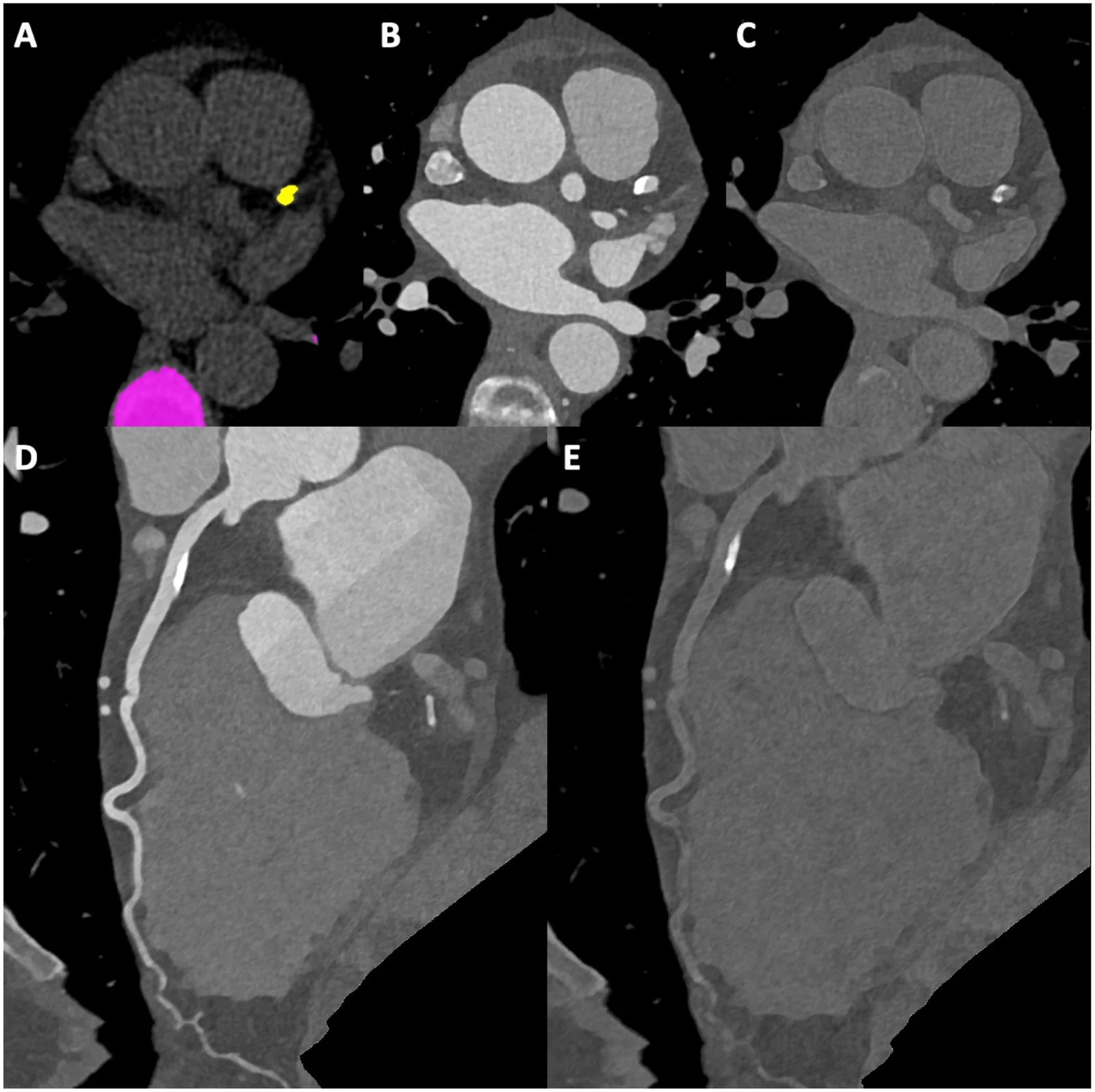
Coronary artery calcium on photon counting CT showing assessment with non-contrast CT (A) and contrast-enhanced CT coronary angiography with virtual mono-energetic image (B, D) and virtual non-contrast image (C, E) Coronary artery calcification is identified in the left anterior descending coronary artery on non-contrast CT (yellow) and contrast-enhanced CT and virtual non-contrast CT (white).

Coronary artery calcium has also emerged as a potential multisystem biomarker of systemic disease and biological aging (Figure 7). The presence and severity of CAC is associated with a wide range of conditions beyond cardiovascular disease and all-cause mortality, including cancer, chronic kidney disease, chronic obstructive pulmonary disease, osteoporosis, dementia, and metabolic disease.^53–56^ However, it must be noted that the correlation between CAC and these different diseases does not demonstrate causation and may be due to shared comorbidities or risk factors. In the MESA, CAC was associated with increased risk of cancer, chronic kidney disease, pneumonia, chronic obstructive pulmonary disease, and hip fracture.^55^ In the Atherosclerosis Risk in Communities (ARIC) study of 2290 individuals over 75 years old without known coronary heart disease, CAC was associated with a variety of markers of aging including dementia, hearing impairment, lung impairment, and grip strength.^56^ The link between CAC and biological aging may be due to a variety of factors such as shared risk factors, shared disease mechanisms including systemic inflammation or oxidative stress, or cardiovascular disease exacerbating other conditions. How this multisystem information should be used in clinical practice is uncertain and is an important subject for future research.

**Figure 7:**
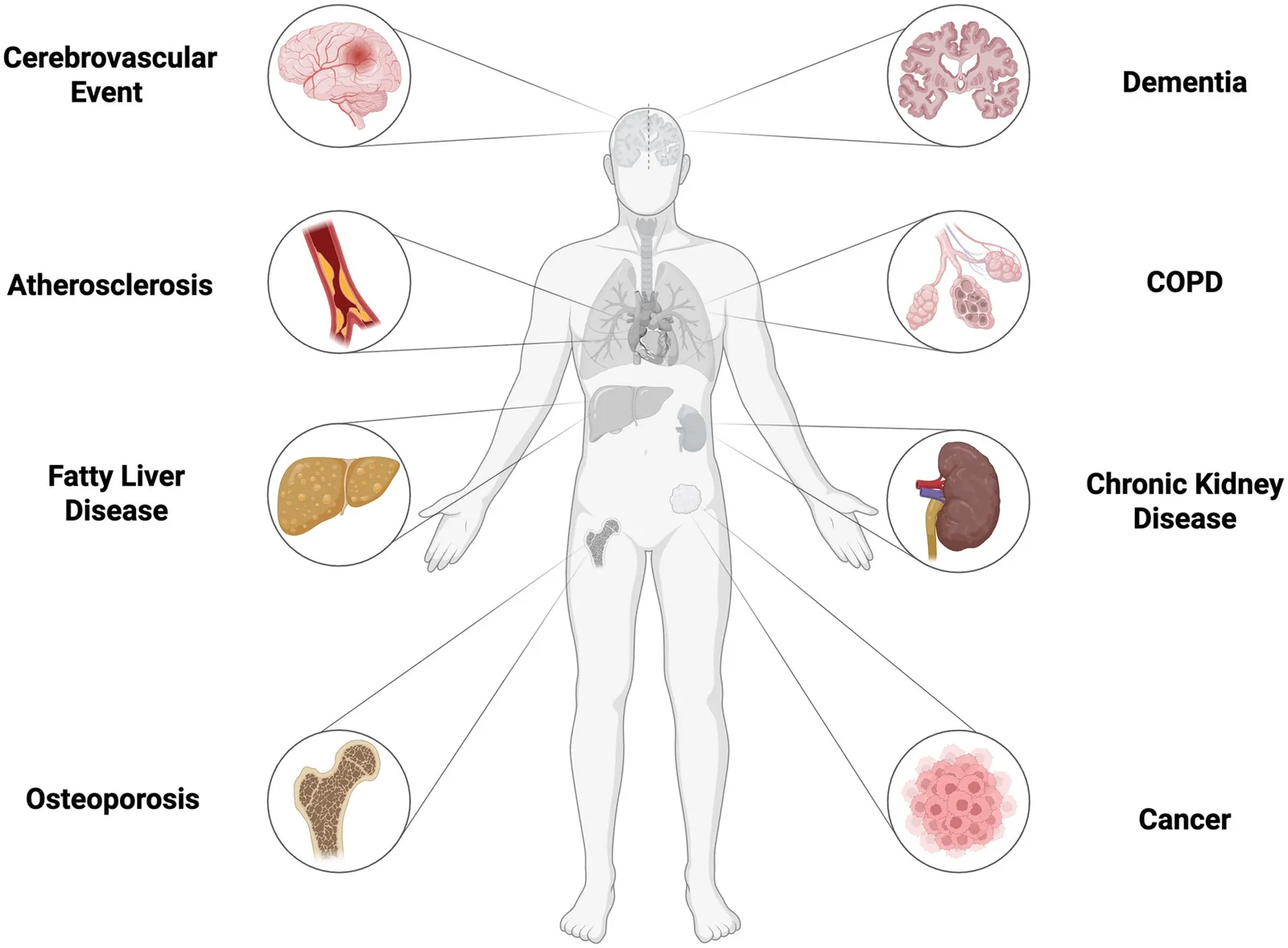
Coronary artery calcification (CAC) as a multi-system biomarker. Correlations between CAC and other diseases have been identified, but this may be due to shared comorbidities or risk factors rather than causation (Created in BioRender. Patel, R. (2025); https://BioRender.com/c1cvpg1). Abbreviation: COPD = chronic obstructive pulmonary disease.

## Conclusion

The identification and quantification of CAC on thoracic CT can act as an opportunistic biomarker of cardiovascular risk. Coronary artery calcium can be identified and quantified on all types of CT imaging, with automated AI/ML methods now available. Opportunistic screening offers an inexpensive way to identify patients at increased risk of cardiovascular events who may benefit from more aggressive management of cardiovascular risk factors and medical therapy such as cholesterol lowering medication. Ongoing randomized controlled trials are investigating whether management based on CAC can improve outcomes for patients. The routine reporting of CAC on thoracic CT can be done today, using visual ordinal scores that are simple and rapid to perform. We know that this information improves lifestyle behaviors and optimizes management, but we eagerly await randomized studies of the impact of these changes on hard cardiovascular outcomes. In the future, AI/ML quantification and analysis tools may automate this process and new techniques such as radiomic analysis and PCCT may further optimize risk stratification.

## Conflicts interest

M.C.W. is supported by the British Heart Foundation (FS/ICRF/20/26002). M.C.W. has given talks for Canon Medical Systems, Siemens Healthineers, GE Healthcare, and Novartis and performed consultancy for FEOPS, Novartis, Bayer, and Canon Medical Systems.
